# Epidermal Growth Factor Gene Polymorphism and Risk of Hepatocellular Carcinoma: A Meta-Analysis

**DOI:** 10.1371/journal.pone.0032159

**Published:** 2012-03-05

**Authors:** Jian-Hong Zhong, Xue-Mei You, Wen-Feng Gong, Liang Ma, Yu Zhang, Qin-Guo Mo, Liu-Cheng Wu, Jun Xiao, Le-Qun Li

**Affiliations:** 1 Hepatobiliary Surgery Department, Guangxi Medical University, Tumor Hospital, Nanning, People's Republic of China; 2 Breast Surgery Department, Guangxi Medical University, Tumor Hospital, Nanning, People's Republic of China; 3 Gastrointestinal Surgery Department, Guangxi Medical University, Tumor Hospital, Nanning, People's Republic of China; The University of Hong Kong, Hong Kong

## Abstract

**Background:**

Hepatocarcinogenesis is a complex process that may be influenced by many factors, including polymorphism in the epidermal growth factor (EGF) gene. Previous work suggests an association between the EGF 61*A/G polymorphism (rs4444903) and susceptibility to hepatocellular carcinoma (HCC), but the results have been inconsistent. Therefore, we performed a meta-analysis of several studies covering a large population to address this controversy.

**Methods:**

PubMed, EMBASE, Google Scholar and the Chinese National Knowledge Infrastructure databases were systematically searched to identify relevant studies. Data were abstracted independently by two reviewers. A meta-analysis was performed to examine the association between EGF 61*A/G polymorphism and susceptibility to HCC. Odds ratios (ORs) and 95% confidence intervals (95% CIs) were calculated.

**Results:**

Eight studies were chosen in this meta-analysis, involving 1,304 HCC cases (1135 Chinese, 44 Caucasian and 125 mixed) and 2,613 controls (1638 Chinese, 77 Caucasian and 898 mixed). The EGF 61*G allele was significantly associated with increased risk of HCC based on allelic contrast (OR = 1.29, 95% CI = 1.16–1.44, p<0.001), homozygote comparison (OR = 1.79, 95% CI = 1.39–2.29, p<0.001) and a recessive genetic model (OR = 1.34, 95% CI = 1.16–1.54, p<0.001), while patients carrying the EGF 61*A/A genotype had significantly lower risk of HCC than those with the G/A or G/G genotype (A/A vs. G/A+G/G, OR = 0.66, 95% CI = 0.53–0.83, p<0.001).

**Conclusion:**

The 61*G polymorphism in EGF is a risk factor for hepatocarcinogenesis while the EGF 61*A allele is a protective factor. Further large and well-designed studies are needed to confirm this conclusion.

## Introduction

As the most frequent primary cancer of the liver, hepatocellular carcinoma (HCC) is the fifth most common solid tumor worldwide and the third leading cause of cancer-related deaths, exceeded only by lung cancer and gastric cancer [1]. The estimated incidence of new HCC cases each year is approximately 500 000–1 000 000, and it causes 600 000 deaths globally each year [1]. In fact, the number of HCC-related deaths nearly equals the number of cases diagnosed each year [2]. The highest incidence rates of HCC (>20 per 100,000) were reported from countries in sub-Saharan Africa and Southeast Asia [2]. Cirrhosis, particularly when it is related to infection by hepatitis C virus (HCV) and/or hepatitis B virus (HBV), is the strongest known risk factor for HCC [3]–[4]. The pathogenesis of HCC may involve chronic inflammation, hepatocyte hyperplasia and ultimately malignant transformation [5]. HCC exhibits a high degree of genetic heterogeneity: multiple molecular pathways may give rise to subsets of hepatocellular neoplasms [5]. For this reason, HCC pathogenesis remains incompletely understood.

Most diagnoses of HCC are made after the disease has progressed substantially. In addition, current therapies for HCC are ineffective for most patients. Consequently, effective screening and chemoprevention depend on early identification of high-risk populations [6]. Traditionally, serum alpha fetoprotein measurement and liver imaging have been the two main strategies for screening high-risk populations. However, both techniques have low sensitivity and specificity, limiting their effectiveness [7]–[9]. For this reason, identification of molecular markers associated with increased risk of HCC would better define high-risk populations of HCC, helping to improve prevention and treatment strategies.

Epidermal growth factor (EGF) has many biological functions involving stimulation of proliferation, differentiation and tumorigenesis of epidermal and epithelial tissues [10]–[11]. EGF is a mitogen for adult and fetal hepatocytes grown in culture, and its expression is up-regulated during liver regeneration [12]. In recent years, numerous studies have associated a single-nucleotide polymorphism involving an A-to-G mutation at position 61 of the 5′ untranslated region of the EGF gene (61*A/G, rs4444903) with the risk of tumorigenesis in multiple human cancers [13]–[15]. This polymorphism modulates tissue-specific EGF gene expression.

In 2008, Tanabe and coworkers [16] explored the association between EGF polymorphism and risk of HCC. They found that the EGF gene polymorphism 61*A/G is associated with risk of developing HCC. Subsequently, epidemiological studies have evaluated the association between the EGF gene polymorphism 61*A/G and risk of HCC in diverse ethnicities [17]–[21]. However, the results have been inconsistent. Some studies have indicated that patients carrying G/G genotypes have a higher susceptibility to HCC [16]–[17], while other studies have not [18]–[20]. A single case-control study may fail to completely demonstrate this complicated genetic relationship because of small sample size. In order to provide strong evidence of the effects of this EGF polymorphism on HCC risk, we carried out a meta-analysis by combining data from numerous published studies.

## Materials and Methods

### Search strategy

All case-control studies of EGF polymorphism and HCC risk published up to October 1, 2011 were identified through systematic searches in PubMed, EMBASE, Google Scholar and the Chinese National Knowledge Infrastructure (CNKI) databases using English and Chinese. The search terms used were: *EGF; epidermal growth factor;* these two terms in combination with *polymorphism*, *variation*, *genotype*, *genetic* and *mutation;* and all of the above terms in combination with *hepatocellular carcinoma*, *HCC*, *liver cancer*, *liver tumor*, *liver neoplasms* and *hepatic tumor*. For each article identified, manual search of the relevant references was also performed.

### Inclusion criteria

A study was included in the meta-analysis if it satisfied the following criteria: (a) it assessed the correlation between HCC and the EGF 61*A/G polymorphism, (b) it used a case-control design, and (c) it provided sufficient published data for estimating an odds ratio (OR) with a 95% confidence interval (95% CI). In the case of multiple studies with the same or overlapping data published by the same researchers, we selected the most recent study with the largest number of participants.

### Data extraction

Literature searches and identification of eligible articles based on the inclusion criteria were carried out independently by two authors (JHZ and XMY). Then each of these authors independently extracted data about the first author's name; year of publication; country of origin; ethnicity, numbers and genotypes of cases and controls; source of controls (hospital- or population-based); frequency of G allele; genotyping method; and Hardy-Weinberg equilibrium (HWE) of controls. Discrepancies were resolved by consensus.

### Statistical methods

The unadjusted OR with 95% CI was used to assess the strength of the association between the EGF 61*A/G polymorphism and HCC based on the genotype frequencies in cases and controls. Subgroup analysis stratified by ethnicity was performed. Ethnicity was categorized as Chinese, mixed or Caucasian. The meta-analysis examined the association of different genotypes at EGF 61*A/G (rs4444903) with HCC risk by comparing the G allele and the A allele (G-allele vs. A-allele), the homozygous genotypes G/G and A/A (G/G vs. A/A), the homozygous genotype (G/G) and recessive genotypes A/A and G/A (G/G vs. A/A+G/A), and the dominant genotype A/A and G/G+G/A (A/A vs. G/G+G/A).

Fixed-effect and random-effect models were used to calculate a pooled OR. The statistical significance of the pooled OR was determined by the Z-test, and P<0.05 was considered statistically significant. The assumption of heterogeneity was evaluated by applying a chi square-based Q-test among the studies. In this approach, the Q value is defined to be identical to the effect size of the chi square. A P value more than 0.10 for the Q-test indicated a lack of heterogeneity, in which case a pooled OR was calculated for each study using the fixed-effects model. Otherwise, the random-effects model was used. Publication bias was assessed by visual inspection of Begg's funnel plots. An asymmetric plot suggested possible publication bias, in which case Egger's test [22] was used. HWE in the control group was assessed using Fisher's exact test, with P<0.05 considered significant. All statistical tests for this meta-analysis were performed using RevMan 5.0 software.

## Results

### Description of studies

**Figure 1:**
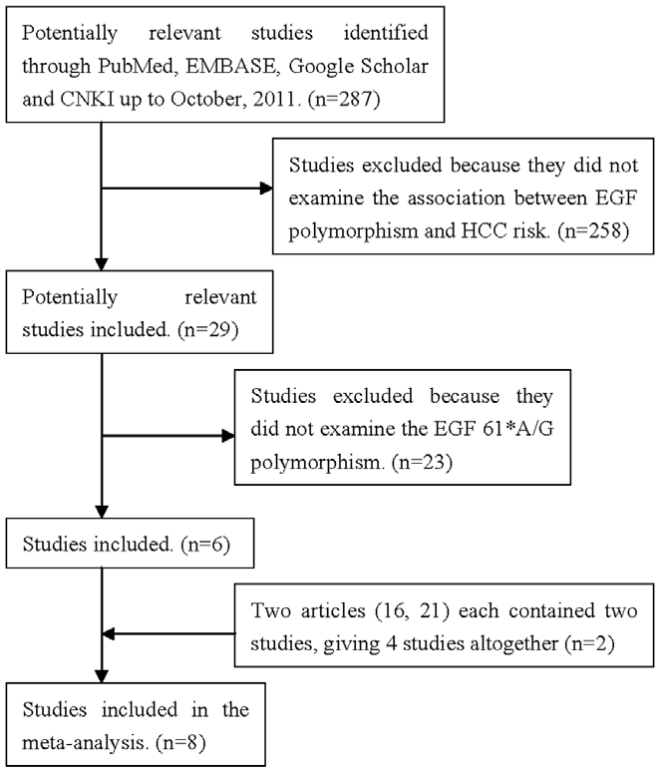
Flow chart of study selection. EGF, epidermal growth factor; HCC, hepatocellular carcinoma.

A total of 287 potentially relevant publications up to October 1, 2011 were systematically identified through PubMed, EMBASE, Google Scholar and CNKI. Of these, 258 (90%) were excluded because they did not satisfy the inclusion criteria, or they failed to provide sufficient information to determine whether the criteria were satisfied. An additional 23 publications were excluded because they did not examine the EGF 61*A/G polymorphism or they were review articles. Two articles reporting a relationship between the EGF 61*A/G polymorphism and chronic hepatitis C [23]–[24] were also excluded, because the participants in these two articles did not have HCC. The article by Kovar et al. [25] investigated the influence of the EGF 61*A/G polymorphism on the recurrence of liver metastases in patients with colorectal cancer and was excluded. Two publications [20], [26] had the same first author and were based on the same participants with HCC, so they were considered as one study. The articles by Tanabe et al. [16] and Wang et al. [21] each involved two independent case-control studies and were considered separately, giving 4 studies altogether. In the end, 8 studies [16]–[21] were included in this meta-analysis based on our search strategy and inclusion criteria (Fig. 1).

We established a database according to the information extracted from each article. Detailed characteristics of the 8 studies are listed in Table 1. Overall, 1,304 HCC cases and 2,613 controls were retrieved. Five of the studies involved Chinese subjects [18]–[21], two involved mixed populations (White, Black, Hispanic, Asian and other) [16]–[17] and one involved Caucasians [16]. All studies had a case-control design, and three [18]–[20] included a healthy control population (population-based control). These three studies [18]–[20] involved 521 HCC cases and 514 controls. The number of cases in the hospital-based control was 2,099. Of the total number of 3,917 subjects considered in the meta-analysis, 3,403 (86.9%) were with cirrhosis and/or infected with hepatitis B virus. The distribution of genotypes among controls showed HWE in all the studies.

**Table 1:** Main characteristics of all studies included in the meta-analysis.

Study	Ethnicity	Genotyping method	Source of control	P_HWE_	Frequency of G allele	Cases/Controls	No. of cases			No. of controls		
							GG	GA	AA	GG	GA	AA
Tanabe 2008a^16^	Mixed	PCR-RFLP	HB	0.19	0.001	59/148	23	27	9	32	65	51
Tanabe 2008b^16^	Caucasian	PCR-RFLP	HB	0.99	0.04	44/77	15	17	12	12	37	28
Abu 2011^17^	Mixed	allele-specific PCR	HB	0.08	0.08	66/750	24	25	17	180	350	220
Chen 2011^18^	Chinese-Han	PCR-RFLP	HB and PB	0.56	0.11	120/240	62	51	7	106	110	24
Li 2009^19^	Chinese-Han	PCR-RFLP	HB and PB	0.94	0.12	186/338	96	82	8	161	145	32
Qi 2009^20^	Chinese-Han	PCR-RFLP	HB and PB	0.75	0.55	215/380	102	98	15	182	160	38
Wang 2009a^21^	Chinese*	PCR-RFLP	HB	0.37	0.06	397/480	200	163	34	209	222	49
Wang 2009b^21^	Chinese-Han	PCR-RFLP	HB	0.53	0.06	217/200	125	76	16	94	89	17

Abbreviations: PCR-RFLP, polymerase chain reaction-restriction fragment length polymorphism; PB, population-based; HB, hospital-based; P_HWE_, Hardy-Weinberg equilibrium of controls.

* Included multiple ethnicities in China.

### Test of heterogeneity

Table S1 shows the relationship between the EGF 61*A/G polymorphism and HCC risk. The heterogeneity of EGF 61*A/G allelic contrast, homozygote comparison, and dominant and recessive genetic models was analyzed for all 8 studies. Random-effect models were used to analyze the OR for the mixed population (G-allele vs. A-allele, G/G vs. A/A, G/G+G/A vs. A/A). Fixed-effect models were used to analyze the OR for the other populations.

### Quantitative data synthesis

Table S1 shows the summary ORs for the EGF 61*A/G polymorphism and HCC risk on the basis of 1,304 HCC cases and 2,613 controls. We observed an association between EGF genotype and HCC risk in the total population based on all 8 studies. Given the ethnic differences in the allele frequency of this sequence variant, we evaluated the effect of EGF 61*A/G polymorphism in Chinese, mixed and Caucasian populations separately. We also evaluated the summary ORs stratified by source of control (hospital- or population-based).

### Total population

**Figure 2:**
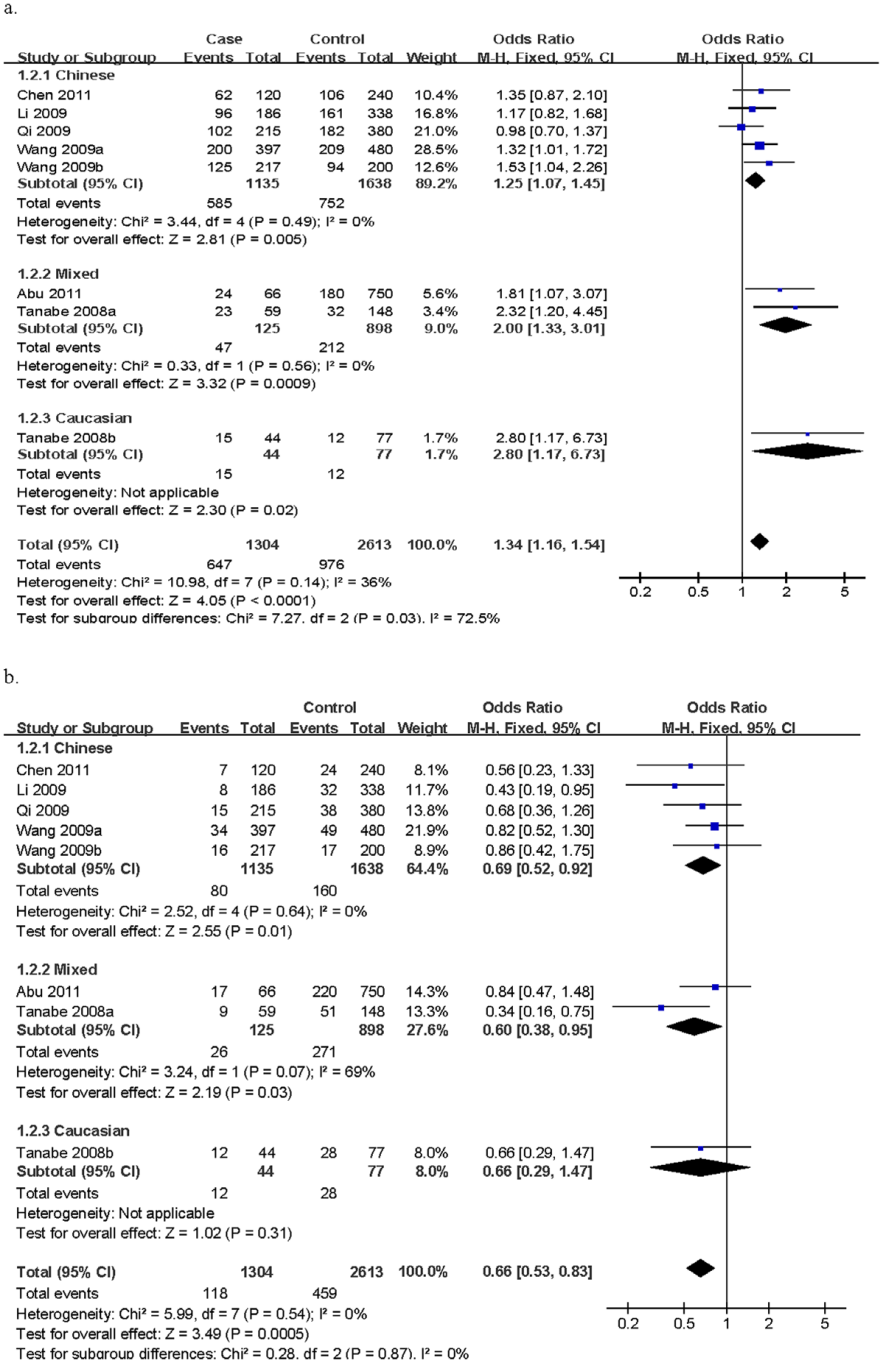
Forest plots describing the association of EGF polymorphism 61*A/G with hepatocellular carcinoma. (a) ORs were calculated by comparing the G/G genotype with the G/A+A/A genotypes. (b) ORs were calculated by comparing the A/A genotype with the G/A+G/G genotypes.

Calculation of overall OR in the total population using the fixed-effect model showed that the 61*G allele was strongly associated with increased risk of HCC in allelic contrast (OR = 1.29, 95% CI = 1.16–1.44, *P*<0.001; *I* ^2^ = 20%), homozygote comparison (OR = 1.79, 95% CI = 1.39–2.29, *P*<0.001; *I* ^2^ = 0%) and the recessive genetic model (OR = 1.34, 95% CI = 1.16–1.54, *P*<0.001; *I* ^2^ = 36%) (Fig. 2. a). Association of the EGF 61*A/A genotype with decreased HCC risk was observed in the total population in the dominant genetic model (OR = 0.66, 95% CI = 0.53–0.83, *P*<0.001; *I* ^2^ = 0%) (Fig. 2. b).

Sensitivity analysis showed that similar results were obtained when a random-effect model was used.

### Ethnicity

#### Chinese population

After stratification for ethnicity, we observed that in the Chinese population, the G-allele, homozygote variant (G/G) and recessive genetic model were significantly associated with increased risk of HCC (G-allele, OR = 1.22, 95% CI = 1.08–1.37, *P* = 0.001; G/G, OR = 1.58, 95% CI = 1.17–2.12, *P* = 0.002; recessive model, OR = 1.25, 95% CI = 1.07–1.45, *P* = 0.005). However, this association was not observed in the dominant genetic model (OR = 0.69, 95% CI = 0.52–0.92, *P* = 0.01).

#### Mixed population

Analysis of the mixed population in two studies revealed that the G-allele, homozygote variant (G/G) and recessive genetic model were significantly associated with increased risk of HCC using a fixed-effect model (G-allele, OR = 1.67, 95% CI = 1.10–2.52, *P* = 0.01; G/G, OR = 2.51, 95% CI = 1.09–5.78, *P* = 0.03; recessive model, OR = 2.00, 95% CI = 1.33–3.01, *P*<0.001). However, the dominant EGF genotype was not associated with HCC risk in these two studies.

#### Caucasian population

The EGF 61*A/G polymorphism was associated with increased risk of HCC among Caucasians (G-allele vs. A-allele, OR = 1.75, 95% CI = 1.03–2.97, *P* = 0.04; G/G vs. A/A, OR = 2.92, 95% CI = 1.06–8.06, *P* = 0.04; recessive model, OR = 2.80, 95% CI = 1.17–6.73, *P* = 0.02). However, the dominant genetic model was not associated with significantly lower HCC risk among Caucasians (OR = 0.66, 95% CI = 0.29–1.47, *P* = 0.31).

### Source of control

#### Hospital-based control

Overall, the variant genotypes G/G+G/A of EGF 61*A/G were associated with significantly higher HCC risk than was the A/A genotype (G/G vs. A/A, OR = 1.87, 95% CI = 1.45–2.42, *P*<0.001; recessive model, OR = 1.47, 95% CI = 1.26–1.71, *P*<0.001). We also found that the frequency of the G allele was strongly associated with increased risk of HCC in allelic contrast (OR = 1.36, 95% CI = 1.21–1.52, *P*<0.001).

#### Population-based control

When comparing population-based controls, we observed an association between the polymorphism and decreased HCC risk in the dominant genetic model (OR = 0.61, 95% CI = 0.38–0.97, *P* = 0.04). Interestingly, the polymorphism was not associated with significantly increased risk in allelic contrast (OR = 1.06, 95% CI = 0.88–1.29, *P* = 0.52), homozygote comparison (OR = 1.56, 95% CI = 0.96–2.55, *P* = 0.07) or the recessive genetic model (OR = 0.97, 95% CI = 0.76–1.23, *P* = 0.78).

### Publication bias

**Figure 3:**
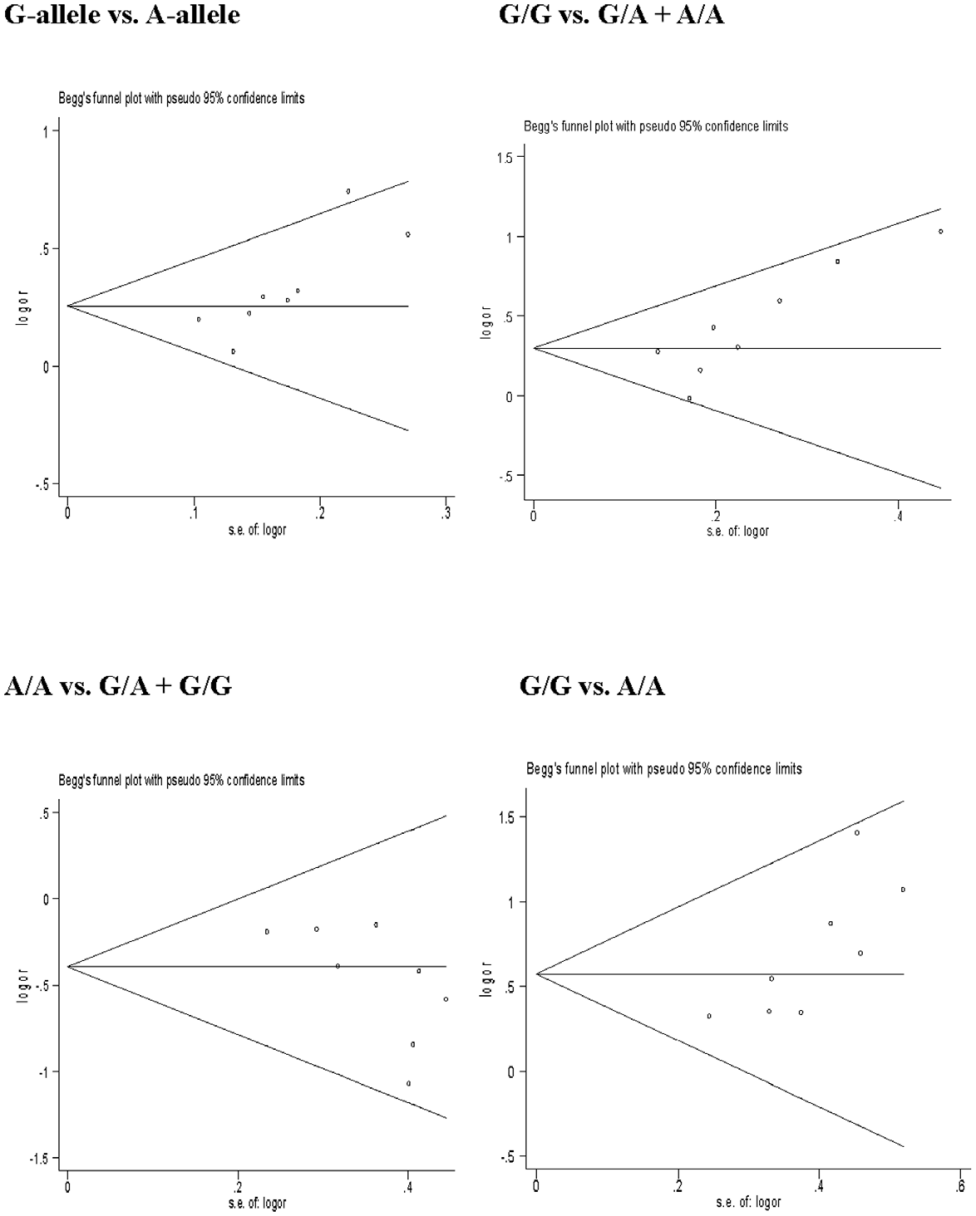
Begg's funnel plots to examine publication bias for reported comparisons of the EGF polymorphism 61*A/G. Plots are shown with pseudo 95% confidence limits. S.E., standard error.

Begg's funnel plots were prepared and Egger's test was performed on the final set of 8 studies [16]–[21] to assess publication bias for reported comparisons of 61*A/G genotypes and HCC. The shape of the funnel plots (Fig. 3) seemed asymmetrical for the comparison of different alleles of the EGF 61*A/G polymorphism, suggesting the presence of publication bias. Therefore, Egger's test was performed to assess funnel plot symmetry statistically. No evidence of publication bias was found for comparisons of EGF 61* A/A with G/G+G/A (*P* = 0.061). However, the funnel plot did show some asymmetry, subsequently corroborated by Egger's test, for comparisons of the EGF 61* G allele and A allele (*P* = 0.021), G/G and A/A (*P* = 0.019), and G/G and G/A+A/A (*P* = 0.023) (Table 2).

**Table 2:** Publication bias tests for comparisons involving the EGF 61*A/G polymorphism.

Genetic comparison	Coefficient	Standard error	t	P value	95% CI of intercept
G-allele vs. A-allele	3.073	0.985	3.12	0.021	0.662 to 5.484
G/G vs. A/A	3.110	0.981	3.17	0.019	0.710 to 5.511
G/G+G/A vs. A/A	−2.766	1.199	−2.31	0.061	−5.700 to 0.168
G/G vs. G/A+A/A	2.889	0.954	3.03	0.023	0.554 to 5.224

## Discussion

One characteristic of tumors is dysregulation of cell growth. HCC involves complex, multistep and heterogeneous malignant tumorigenesis. The pathogenesis of HCC involves host genetic factors, environmental factors and modulation of molecular signaling pathways implicated in malignant transformation of hepatocytes and tumor progression [27]. Cirrhosis associated with HBV and/or HCV infection and alcohol is the most well established environmental risk factor for HCC around the world. In fact, cirrhosis is considered a precancerous stage to some extent, although only a fraction of cirrhosis patients and HCV-infected individuals develop HCC later in life [28]. Moreover, some patients without known risk factors eventually develop HCC [29]. Therefore, genetic predisposition may contribute to the process of hepatocarcinogenesis.

Many meta-analyses have shown that polymorphism in some genes strongly correlates with susceptibility to HCC [30]–[32]. Some studies have reported an association between polymorphism in EGF 61*A/G and HCC risk [16]–[17]. The EGF receptor signaling pathway is thought to be an important mediator of hepatocyte proliferative capacity and liver regeneration as a result of chronic liver injury [33]. Dysregulation of the EGF receptor signaling pathway plays an important role in early hepatocarcinogenesis and other tumorigenesis [34]–[36]. One mechanism by which the EGF gene polymorphism may lead to increased risk of HCC is by modulating EGF levels.

Other studies of the EGF 61*A/G polymorphism and HCC risk failed to find an association (Table 3). The most likely reason for the inconsistencies among these studies is that most are single case-control studies with small sample sizes. To help resolve these conflicting results using a larger sample size, we conducted meta-analysis of published studies. Our results for the total population suggest an increased HCC risk for subjects carrying the EGF 61*G/G genotype, and a protective effect for the A/A genotype. Our approach also allowed us to look for potential ethnic differences in the association. Analysis of ethnic subgroups showed that in the three different groups (Chinese, Caucasian and mixed), the 61*G allele was highly associated with increased risk of HCC based on allelic contrast, homozygote comparison and the recessive genetic model. Our findings are in line with those of a recently published meta-analysis showing that the EGF 61*G/G genotype in Caucasians is associated with increased risk of glioma [37], and recurrence of liver metastases [25].

**Table 3:** Odds ratios and 95% confidence intervals reported for the association between hepatocellular carcinoma and EGF 61*A/G genotype.

Study	Genotype comparison			
	G-allele vs. A-allele	G/G vs. A/A	G/G vs. G/A+A/A	A/A vs. G/G+G/A
Tanabe 2008a^16^	2.10 (1.36–3.25)	4.07 (1.68–9.90)	2.32 (1.20–4.45)	0.34 (0.16–0.75)
Tanabe 2008b^16^	1.75 (1.03–2.97)	2.92 (1.06–8.06)	2.80 (1.17–6.73)	0.66 (0.29–1.47)
Abu 2011^17^	1.38 (0.96–1.97)	1.73 (0.90–3.31)	1.81 (1.07–3.07)	0.84 (0.47–1.48)
Chen 2011^18^	1.32 (0.94–1.86)	2.01 (0.82–4.92)	1.35 (0.87–2.10)	0.56 (0.23–1.33)
Li 2009^19^	1.25 (0.94–1.66)	2.39 (1.06–5.39)	1.17 (0.82–1.68)	0.43 (0.19–0.95)
Qi 2009^20^	1.06 (0.82–1.37)	1.42 (0.74–2.71)	0.98 (0.70–1.37)	0.68 (0.36–1.26)
Wang 2009a^21^	1.22 (0.99–1.49)	1.38 (0.85–2.23)	1.32 (1.01–1.72)	0.82 (0.52–1.30)
Wang 2009b^21^	1.34 (0.99–1.82)	1.41 (0.68–2.94)	1.53 (1.04–2.26)	0.86 (0.42–1.75)

Our meta-analysis suggests that the EGF 61*A polymorphism may reduce susceptibility to HCC among Chinese. For mixed or Caucasian populations, however, our stratified meta-analysis according to ethnicity failed to demonstrate a statistically significant protection from HCC associated with the EGF 61*A homozygous variant genotype. This is most probably because our meta-analysis involved only two studies with a mixed population and only one with a Caucasian population. There may be a high risk of selective bias for the relationship between the EGF 61*G/A polymorphism and HCC development in these two populations, so this association should be re-evaluated in studies with large sample sizes. Therefore, the negative results in the present study should be interpreted with caution.

All the patients in the hospital-based control populations had HBV infection and/or cirrhosis. Among these hospital-based controls, the EGF 61*G allele was statistically associated with increased risk of HCC based on allelic contrast, homozygote comparison and the recessive genetic model. In contrast, this polymorphism was associated with decreased HCC risk in the dominant genetic model in both the hospital- and population-based control populations. At the same time, the EGF 61*G allele was not associated with HCC susceptibility in the population-based control. The G/G genotype frequency in the case group was significantly higher than in the control group with HBV infection and/or cirrhosis, implying that HBV and/or cirrhotic patients with the G/G genotype may be at higher risk of HCC development. At the same time, the frequency of the G/G genotype in healthy individuals (261/514) was similar to that in the case group (260/521), suggesting that healthy individuals with the G/G genotype may not be at higher risk of developing HCC. We infer from these results that environmental factors are more important than host genetic factors in HCC. In other words, environmental factors mediate the ability of genetic factors to contribute to HCC. Our findings suggest that the EGF 61*A/G polymorphism is a genetic susceptibility factor for HCC only in the background of chronic HBV infection and/or cirrhosis.

Since ethnicities can show different genotype frequencies, ethnicity should be taken into account in genetic association studies. Previous studies [19]–[20] have found the frequency of the EGF 61*A/A genotype to be approximately 10% in Asians but approximately 30% in Caucasians. Similarly, the present study found a higher frequency of the A/A genotype in the mixed and Caucasian populations than in the Chinese population. The pooled ORs of this meta-analysis suggest that the EGF 61*G allele is a risk factor for HCC, while the EGF 61*A allele is a protective factor. Therefore, differences in the distribution of EGF genotypes among different ethnicities may help explain the higher HCC prevalence among Asians [2].

Some limitations of this meta-analysis should be considered when interpreting the results. One of the major concerns is bias due to selective publication. Obvious publication bias was detected in the comparison of G- and A-alleles, G/G and A/A genotypes, and G/G and G/A+A/A genotypes. Second, bias may result from the fact that unpublished data, as well as papers published in languages other than English and Chinese, were not included. Third, the results may be affected by additional confounding factors, such as hepatitis B infection status, tumor status, gender or age, but most studies either did not report these baseline data or aggregated them in different ways, making it impossible to include them in the meta-analysis. Fourth, the number of published studies included in our meta-analysis was not sufficiently large for a comprehensive analysis. In particular, the subgroup analyses of a mixed-ethnic population and a Caucasian population were based on only two and one study, respectively. Fifth, there was significant heterogeneity among the studies in the different ethnic subgroups. Sixth, nearly all of the studies were performed in Asian and Caucasian populations; further studies are needed in other ethnic groups in order to capture the full range of possible ethnic differences in EGF polymorphisms.

In conclusion, this meta-analysis suggests that the G-allele of the EGF 61* polymorphism (rs4444903) is associated with increased risk of HCC, while the A-allele contributes to decreased susceptibility to HCC, especially in the Chinese population. These results suggest that EGF gene variation may play an important role in the occurrence of HCC. However, since this meta-analysis included few studies from non-Asian populations, large, well-designed studies in Caucasian and African-American populations are warranted to re-evaluate these associations.

This meta-analysis is guided by the PRISMA statement (Checklist S1).

## Supporting Information

Table S1 Overall and stratified meta-analysis of the association between the EGF 61*A/G polymorphism and HCC risk.
(DOC)

Checklist S1 **PRISMA 2009 Checklist.**
(DOC)
