# Supplementary material for: Epidermal Growth Factor Gene Polymorphism and Risk of Hepatocellular Carcinoma: A Meta-Analysis
Source: PLoS One. 2012 Mar 5;7(3):e32159. doi: 10.1371/journal.pone.0032159 (PMC3293888; doi:10.1371/journal.pone.0032159)
Supplement: Table S1 — Overall and stratified meta-analysis of the association between the EGF 61*A/G polymorphism and HCC risk. (DOC) [file pone.0032159.s001.doc]

**Table S1.** Overall and stratified meta-analysis of the association between the EGF 61*A/G polymorphism and HCC risk.

| Genotype comparison | OR [95% CI] | Z (P value) | Heterogeneity of study design | | | Analysis model |
| --- | --- | --- | --- | --- | --- | --- |
|  |  |  | χ^2^ | df (P value) | I^2^ |  |
| **Total (1304 cases, 2613 controls)** | | | | | | |
| G-allele vs. A-allele | 1.29 [1.16, 1.44] | 4.70 (＜0.001) | 8.78 | 7 (0.27) | 20% | Fixed |
| G/G vs. A/A | 1.79 [1.39, 2.29] | 4.58 (＜0.001) | 6.76 | 7 (0.45) | 0% | Fixed |
| G/G vs. G/A+A/A | 1.34 [1.16, 1.54] | 4.05 (＜0.001) | 10.98 | 7 (0.14) | 36% | Fixed |
| A/A vs. G/G+G/A | 0.66 [0.53, 0.83] | 3.49 (＜0.001) | 5.99 | 7 (0.54) | 0% | Fixed |
| **Ethnic subgroups** | | | | | | |
| **Chinese (1135 cases, 1638 controls)** | | | | | | |
| G-allele vs. A-allele | 1.22 [1.08, 1.37] | 3.27 (0.001) | 1.71 | 4 (0.79) | 0% | Fixed |
| G/G vs. A/A | 1.58 [1.17, 2.12] | 3.03 (0.002) | 1.75 | 4 (0.78) | 0% | Fixed |
| G/G vs. G/A+A/A | 1.25 [1.07, 1.45] | 2.81 (0.005) | 3.44 | 4 (0.49) | 0% | Fixed |
| A/A vs. G/G+G/A | 0.69 [0.52, 0.92] | 2.55 (0.01) | 2.52 | 4 (0.64) | 0% | Fixed |
| **Mixed (Caucasian, Chinese and Black) (125 cases, 898 controls)** | | | | | | |
| G-allele vs. A-allele | 1.67 [1.10, 2.52] | 2.43 (0.01) | 2.15 | 1 (0.14) | 53% | Random |
| G/G vs. A/A | 2.51 [1.09, 5.78] | 2.16 (0.03) | 2.33 | 1 (0.13) | 57% | Random |
| G/G vs. G/A+A/A | 2.00 [1.33, 3.01] | 3.32 (＜0.001) | 0.33 | 1 (0.56) | 0% | Fixed |
| A/A vs. G/G+G/A | 0.56 [0.23, 1.33] | 1.31 (0.19) | 3.24 | 1 (0.07) | 69% | Random |
| **Caucasian (44 cases, 77 controls)** | | | | | | |
| G-allele vs. A-allele | 1.75 [1.03, 2.97] | 2.07 (0.04) |  |  |  |  |
| G/G vs. A/A | 2.92 [1.06, 8.06] | 2.06 (0.04) |  |  |  |  |
| G/G vs. G/A+A/A | 2.80 [1.17, 6.73] | 2.30 (0.02) |  |  |  |  |
| A/A vs. G/G+G/A | 0.66 [0.29, 1.47] | 1.02 (0.31) |  |  |  |  |
| **Source of control** | | | | | | |
| **Hospital-based (1304 cases, 2099 controls)** | | | | | | |
| G-allele vs. A-allele | 1.36 [1.21, 1.52] | 5.33 (＜0.001) | 7.75 | 7 (0.36) | 0% | Fixed |
| G/G vs. A/A | 1.87 [1.45, 2.42] | 4.77 (＜0.001) | 7.38 | 7 (0.39) | 0% | Fixed |
| G/G vs. G/A+A/A | 1.47 [1.26, 1.71] | 4.95 (＜0.001) | 7.94 | 7 (0.34) | 12% | Fixed |
| A/A vs. G/G+G/A | 0.66 [0.52, 0.84] | 3.41 (＜0.001) | 6.35 | 7 (0.50) | 0% | Fixed |
| **Population-based (521 cases, 514 controls)** | | | | | | |
| G-allele vs. A-allele | 1.06 [0.88, 1.29] | 0.64 (0.52) | 0.30 | 2 (0.86) | 0% | Fixed |
| G/G vs. A/A | 1.56 [0.96, 2.55] | 1.79 (0.07) | 0.71 | 2 (0.70) | 0% | Fixed |
| G/G vs. G/A+A/A | 0.97 [0.76, 1.23] | 0.27 (0.78) | 0.22 | 2 (0.90) | 0% | Fixed |
| A/A vs. G/G+G/A | 0.61 [0.38, 0.97] | 2.07 (0.04) | 0.71 | 2 (0.70) | 0% | Fixed |
